# Prognostic value of mean glycemia and glycemic variability in medical, surgical, and cardiovascular intensive care units at a Lebanese tertiary care center

**DOI:** 10.3389/fendo.2025.1682970

**Published:** 2025-10-10

**Authors:** Rachad Abou Daher, Christy Salameh, Nada El Ghorayeb, Maissa Safieddine, Marie-Hélène Gannagé-Yared

**Affiliations:** ^1^ Department of Endocrinology at Hotel-Dieu de France Hospital, Faculty of Medicine, Saint-Joseph University, Beirut, Lebanon; ^2^ Department of Statistics at Saint-Joseph University, Beirut, Lebanon

**Keywords:** mean glycemia, glycemic variability, intensive care unit, length of stay, mortality

## Abstract

**Background and objectives:**

Stress-induced hyperglycemia is common in intensive care units (ICUs) and has been linked to adverse outcomes. Although mean glycemia (MG) has been extensively studied, the benefits of strict glycemic control remain controversial, and the impact of glycemic variability (GV) is less clearly defined. No consensus currently exists regarding GV thresholds, and limited evidence is available across different ICU settings, with data from the Middle East region particularly lacking. This study aimed to assess the relationship between MG and GV with key clinical outcomes, including hospital and ICU length of stay (LOS), renal function, and in-hospital mortality, among patients admitted to three ICUs at a Lebanese tertiary care center.

**Methods:**

We retrospectively reviewed the medical records of patients admitted during July and August 2024 to the surgical, medical, and cardiovascular ICUs at the Hôtel-Dieu de France Hospital. Baseline characteristics, MG, GV, total hospital/ICU LOS, in-hospital mortality, and glomerular filtration rates (GFR) were analyzed.

**Results:**

GV was significantly associated with prolonged total and ICU LOS, reduced GFR, and increased in-hospital mortality. Patients with GV >30% had a markedly higher risk of death. In contrast, no significant association was found between MG and said outcomes. MG differed across ICU subunits, reaching its highest levels in the cardiovascular ICU, while GV did not vary significantly between units.

**Conclusion:**

GV, rather than MG, emerged as a key predictor of adverse outcomes in ICU patients, being associated with longer hospital and ICU LOS, renal impairment, and increased mortality. These findings highlight GV as an important therapeutic target in the management of critically ill patients.

## Introduction

Stress-induced hyperglycemia occurs in a variety of pathological conditions. Although initially serving as a protective physiological response (1), it proves detrimental when prolonged. This transient rise in blood glucose is triggered by excessive counter-regulatory hormones (e.g., glucagon, catecholamines) and inflammatory cytokines (e.g., IL-1, IL-6, TNF-α), and is further aggravated by insulin resistance (1, 2).

In-hospital hyperglycemia is reported in approximately 38% of hospitalized patients, of whom 26% have a known history of diabetes, while 12% are newly diagnosed (3). It is associated with increased morbidity and mortality (3, 4), particularly among patients without previously diagnosis diabetes (16% vs 3%) (3). Stress-induced hyperglycemia has been linked to a higher risk of infections (5), increased mortality after myocardial infarction (6), and poorer outcomes in patients with stroke (7) or traumatic brain injury (8). The role of strict glycemic control in the ICU remains highly controversial. Some studies (9–11) have reported improved outcomes with intensive insulin therapy, whereas others, including VISEP (12) and CREATE-ECLA (13)) found no significant benefit and even indicated potential harm (14). The large international randomized NICE-SUGAR trial (14) found that stringent glucose control in ICU patients (with an 81-108 mg/dL target) increased mortality and the risk of severe hypoglycemia compared with a more permissive target (<180 mg/dL). Two meta-analyses further contributed to this debate: Pittas et al. (15) reported reduced mortality with strict glycemic control, while Wiener et al. (16) observed improvements only in infection-related outcomes.

Consequently, the American Diabetes Association (ADA) recommends maintaining blood glucose levels between 140 and 180 mg/dL in adult patients admitted to medical and surgical ICUs, as this approach reduces complications during hospitalization (17). Tighter targets (110–140 mg/dL) may be considered in select cases, such as critically ill patients undergoing cardiac surgery (17).

Beyond MG levels, glycemic variability (GV) has emerged as a potential determinant of in-hospital outcomes. A meta-analysis of 11 studies demonstrated that patients with high GV were at increased risk of cardiovascular events (18). Another recent meta-analysis (19) suggested that GV is a prognostic factor for mortality, partly through its association with ventricular arrhythmias (20). Several recent studies have also explored the relationship between GV and hospital and ICU length of stay (LOS) (21, 22).

Despite the growing body of evidence linking both MG and GV to adverse outcomes, substantial knowledge gaps remain. No universal threshold for GV has been established, and little is known about differences in glycemic control across various ICU settings. Furthermore, data from the Middle East region, particularly from Lebanon, is lacking. To our knowledge, this is the first study to simultaneously examine the impact of MG and GV on multiple outcomes across different ICU subunits within a major tertiary hospital. We hypothesized that higher MG and greater GV would be associated with poorer clinical outcomes in ICU patients. By addressing these gaps, our study aims to provide novel insights into the prognostic value of glycemic patterns in critically ill populations.

## Methods

This retrospective observational study included all patients admitted to the medical, surgical, and cardiovascular ICUs at Hôtel-Dieu de France (HDF) hospital who underwent capillary blood glucose (CBG) monitoring during their stay. Because CBG monitoring was systematically performed in all ICU patients, no exclusion criteria were applied. We reviewed the medical records of patients hospitalized at the ICUs during July and August 2024 and collected the following demographic, clinical, and biological data: age, gender, weight in kilograms (kg), height in meter (m), history of diabetes or hypertension (HTN), corticosteroid or vasopressor uses, total hospital LOS, ICU LOS, in-hospital mortality, and hospital readmission within six months after discharge. A prior history of diabetes or HTN was determined from patient records and medication lists at admission. Body mass index (BMI) was calculated using the formula: weight (kg)/height² (m²). CBG and GFR values were also retrieved. CBG measurements were routinely performed for all ICU patients, with a frequency ranging from 3 to 12 measurements per day depending on clinical needs. MG was calculated for each patient as the arithmetic mean of all CBG values (sum of values/number of values). GV was assessed using the coefficient of variation (CV), expressed as a percentage: CV (%) = (Standard Deviation of blood glucose/Mean blood glucose) × 100. Because this study was retrospective, glucose monitoring frequency was not standardized. To account for this, we used the CV to describe glucose variability, calculated as the ratio of the standard deviation to the mean of all available measurements. For each patient, CBG was categorized into 5 categories as follows:<70 mg/dL; 70–99 mg/dL; 100–139 mg/dL; 140–180 mg/dL; >180 mg/dL. GFR was calculated using the CKD-EPI formula, and expressed in mL/min. GFR was classified according to the 2024 KDIGO guidelines (23) into the following categories: ≥90 mL/min: normal or high GFR; 60–89 mL/min: mild decrease in GFR; 45–59 mL/min: mild to moderate decrease in GFR; 30–44 mL/min: moderate to severe decrease in GFR; 15–29 mL/min: severe decrease in GFR; <15 mL/min: kidney failure. The CKD-EPI formula was defined as: GFR = 141×(min(κScr​,1))α×(max(κScr,1))−1.209×(0.993) Age×[1.018 if female], Where: κ = 0.7 (female), 0.9 (male) and α = –0.329 (female), –0.411 (male).

### Statistical analysis

Descriptive analyses were conducted to summarize clinical and demographic characteristics, as well as patient outcomes. The normality of continuous variables was assessed using the Kolmogorov–Smirnov (KS) and Shapiro–Wilk (SW) tests. Normally distributed variables are presented as mean ± standard deviation, whereas non-normally distributed variables are presented as median (Q1–Q3). Categorical variables are expressed as counts and percentages.

For comparisons of continuous variables between two groups, Student’s t-test was used when the distribution was normal, and the Mann–Whitney U test was applied when the distribution was not normal. For comparisons involving three or more groups, one-way ANOVA was used for normally distributed variables, while the Kruskal–Wallis test was used for non-normally distributed variables. For categorical variables, comparisons between groups were performed using the Chi-squared test, when the conditions for validity were met; otherwise, Fisher’s exact test was applied.

Correlations between variables were evaluated using Spearman’s rank correlation coefficient. To explore trends in GV, several thresholds were assessed, and a 30% threshold was selected *a priori* to define high GV. A corresponding binary variable was created. A Cox proportional hazards regression model was then used to assess the association between high GV and in-hospital-mortality, adjusting for age, sex, type of ICU, diabetes mellitus, hypertension, and systemic corticosteroid exposure. All statistical tests were two-sided, and a p-value of less than 0.05 was considered statistically significant. Statistical analyses were performed using R software version 4.2.2 (packages prettyR, tableone, Epi and Survival).

## Results

### Clinical and anthropometric variables in the study population, by ICU, and by gender

During the study period, 130 patients were admitted to the ICUs: 35 to the medical ICU, 46 to the surgical ICU, and 49 to the cardiovascular ICU. Their demographic and clinical characteristics are presented in **Table 1**. The median age of the overall cohort was 63.5 years [52.25-71], with 51.5% of patients being men. The median MG was 129.9 mg/dL [117.2-151.9], and median GV was 18% (12–26). When stratified by ICU subunit (**Table 2**), age was significantly higher in the cardiovascular ICU (p=0.031). Significant differences were also observed across the three ICUs for BMI (p = 0.041), history of HTN (p = 0.001), and corticosteroid use (p <0.001). When comparing according to gender, men had a significantly higher prevalence of pre-existing HTN (p = 0.03), whereas women showed significantly higher corticosteroid use (p = 0.03). No significant differences were observed by gender for BMI.

**Table 1:** Demographic and biological characteristics of the total population.

Patient characteristics		Population (n=130)
Age (years) (Median [IQR])		63.5 [52.25-71]
Gender: Male (%) Female (%)		67 (51.5) 63 (48.5)
BMI (kg/m^2^) (Median [IQR])		25.50 [23.01-29.07]
Length of stay (days) (Median [IQR])		9.00 [7.00-14.00]
Length of stay in ICU (days) (Median [IQR])		3.00 [2.00-4.00]
Previous Diabetes (%)		30 (23.10%)
Previous Hypertension (%)		75 (57.7%)
Death (%)		12 (9.20%)
Re-hospitalization (%)		36 (27.70%)
Glucocorticoid use (%)		38 (29.20%)
Vasopressors use (%)		28 (21.50%)
Mean Glycemia (mg/dL) (Median [IQR])		129.9 [117.2-151.9]
Percentage of Glycemic Variability (Median [IQR])		18.00 [12.00-26.00]
Number of CBG during the ICU stay (Median [IQR])		13.00 [7.00-20.00]
Percentage of CBG < 70 (mean± SD)		1.59 ± 7.26
Percentage of CBG between [70;100[(mean± SD)		13.33 ± 21.23
Percentage of CBG between [100;140[(mean± SD)		47.81 ± 29.67
Percentage of CBG between [140,180] (mean± SD)		25.83 ± 24.2
Percentage of CBG > 180 (mean± SD)		11.44 ± 19.08
GFR (ml/min) (Median [IQR])		89.5 [58.5-104.8]
GFR Categories (ml/mn) (%)	≥90	65 (50%)
	[60;90]	33 (25.4%)
	[45;59[	7 (5.4%)
	[30;44[	6 (4.6%)
	[15;29[	9 (6.9%)
	<15	10 (7.7%)

SD, standard deviations.

CBG, Capillary blood glucose.

GFR, Glomerular filtration rate.

**Table 2:** Patient characteristics by ICU type.

Variable	Medical ICU (n=35)	Surgical ICU (n=46)	Cardiac ICU (n=49)	P-value
Age (years) (Median [IQR])	64.00 [54.00-76.00]	57.50 [50.00-68.50]	68.00 [58.00-72.00]	0.031
Gender: Male (%) Female (%)	22 (62.9%) 13 (37.1%)	9 (19.6%) 37 (80.4%)	36 (73.5%) 13 (26.5%)	<0.001
BMI (kg/m^2^) (Median [IQR])	24.09 [19.32-27.59]	26.24 [23.21-30.38]	25.69 [23.94-29.38]	0.041
Total Hospital LOS (days) (mean± SD)	10.00 (7.00-17.00)	8.50 (6.00-14.00)	8.00 [7.00-12.00]	0.098
ICU LOS (days) (Median [IQR])	3.00 [2.00-4.00]	3.00 [2.00-5.00]	3.00 [3.00-4.00]	0.626
Previous Diabetes (%)	7 (20%)	7 (15.20%)	16 (32.7%)	0.115
Previous Hypertension (%)	22 (62.9%)	17 (37.00%)	36 (73.5%)	0.001
Death (%)	9 (25.70%)	1 (2.20%)	2 (4.10%)	<0.001
Re-hospitalization (%)	10 (28.60%)	14 (30.40%)	12 (24.50%)	0.804
Corticosteroids use (%)	16 (45.70%)	19 (41.30%)	3 (6.10%)	<0.001
Vasopressors use (%)	12 (34.30%)	6 (13.00%)	10 (20.40%)	0.068

### Biological variables, by ICU, and by gender

During their ICU stay, 79.2% of patients developed hyperglycemia (defined as at least one CBG > 140 mg/dL), with observed rates of 74.3%, 71.7%, and 89.8% in the medical, surgical, and cardiovascular ICUs, respectively. Although these differences were not statistically significant, a trend toward higher rates in the cardiovascular ICU was noted (p = 0.067). The distribution of biological variables according to ICU type is summarized in **Table 3**. Median MG levels differed among ICU subunits, with the highest values observed in the cardiovascular ICU (140.69 mg/dL), compared to 124.66 mg/dL in the medical ICU and 127.30 mg/dL in the surgical ICU (p = 0.011). In contrast, no significant differences were observed in median GV levels (medical ICU: 17%, surgical ICU: 20%, cardiovascular ICU: 16%). A significant difference in GFR was noted across the three different ICUs (p < 0.001). Comparisons by gender showed no significant differences in MG or in GV (p = 0.3 and p = 0.7, respectively) (**Table 4**).

**Table 3:** Distribution of biological variables according to ICUs.

Biologic variable		Medical ICU (n=36)	Surgical ICU (n=46)	Cardiac ICU (n=49)	P-value
Mean Glycemia (mg/dL) (Median [IQR])		124.66 [109.22-141.38]	127.30 [112.99-146.36]	140.69 [125.40-162.80]	0.011
Glycemic Variability (%) (Median [IQR])		17.00 [12.00-33.00]	20.00 [9.00-25.00]	16.00 [12.00-25.00]	0.72
Number of CBG values during ICU stay (Median [IQR])		11.00 [6.50-14.00]	9.00 [5.00-17.75]	17.00 [13.00-22.00]	0.001
Percentage of CBG<70 (mean± SD)		1.27 ± 3.99	3 ± 11.47	0.49 ± 1.96	0.231
Percentage of CBG between [70;100[(mean± SD)		19.58 ± 27.03	13.54 ± 17.6	8.67 ± 18.78	0.066
Percentage of CBG between [100;140[(mean± SD)		49.23 ± 29.76	52.16 ± 30.1	42.71 ± 29.03	0.286
Percentage of CBG between [140;180] (mean± SD)		19.97 ± 17.76	20.06 ± 25.06	35.44 ± 24.71	0.0321
Percentage of CBG > 180 (mean± SD)		9.94 ± 18.36	11.24 ± 20.77	12.71 ± 18.22	0.598
GFR (ml/min(Median [IQR])		63.00 [16.50-96.50]	97.50 [90.25-110.75]	82.00 [60.00-97.00]	<0.001
GFR Categories (ml/mn) (%)	≥90	12 (34.3%)	35 (76.1%)	18 (36.7%)	<0.001
	[60;90]	7 (20.0%)	6 (13.0%)	20 (40.8%)	
	[45;59[	0 (0.0%)	1 (2.2%)	6 (12.2%)	
	[30;44[	3 (8.6%)	1 (2.2%)	2 (4.1%)	
	[15;29[	5 (14.3%)	3 (6.5%)	1 (2.0%)	
	<15	8 (22.9%)	0 (0.0%)	2 (4.1%)	

SD, standard deviations.

CBG, Capillary blood glucose.

GFR, Glomerular filtration rate.

**Table 4:** Comparison of biological variables by gender.

Biological variable		Men (n=67)	Women(n=63)	P-value
Mean Glycemia (mg/dL) (Median [IQR])		133.07 [121.69, 155.62]	127.65 [114.68, 147.30]	0.305
Glycemic variability (%) (Median [IQR])		0.16 [0.12, 0.25]	0.19 [0.11, 0.28]	0.718
Number of CBG values (Median [IQR])		14.00 [9.50, 20.00]	11.00 [5.00, 19.00]	0.162
Percentage of CBG <70 (mean± SD)		1.14 ± 3.46	2.06 ± 9.83	0.47
Percentage of CBG between [70;100[(mean± SD)		12.18 ± 22.7	14.55 ± 19.6	0.53
Percentage of CBG between [100;140[(mean± SD)		45.47 ± 29.5	50.3 ± 29.9	0.35
Percentage of CBG between [140;180] (mean± SD)		29.85 ± 24.22	21.74 ± 23.9	0.06
Percentage of CBG> 180 (mean± SD)		11.53 ± 17.0	11.35 ± 21.2	0.96
GFR (ml/min) (Median [IQR])		79.00 [51.00-97.00]	95.00 [68.00-108.00]	0.031
GFR Categories (ml/mn) (%)	>90	24 (35.8%)	41 (65.1%)	0.001
	[60;90]	25 (37.3%)	8 (12.7%)	
	[45;59[	4 (6.0%)	3 (4.8%)	
	[30;44[	1 (1.5%)	5 (7.9%)	
	[15;29[	5 (7.5%)	4 (6.3%)	
	<15	8 (11.9%)	2 (3.2%)	

SD, standard deviations.

CBG, Capillary blood glucose.

GFR, Glomerular filtration rate.

### Correlation between MG and GV with total hospital and ICU LOS, mortality, and GFR

**Figure 1:**
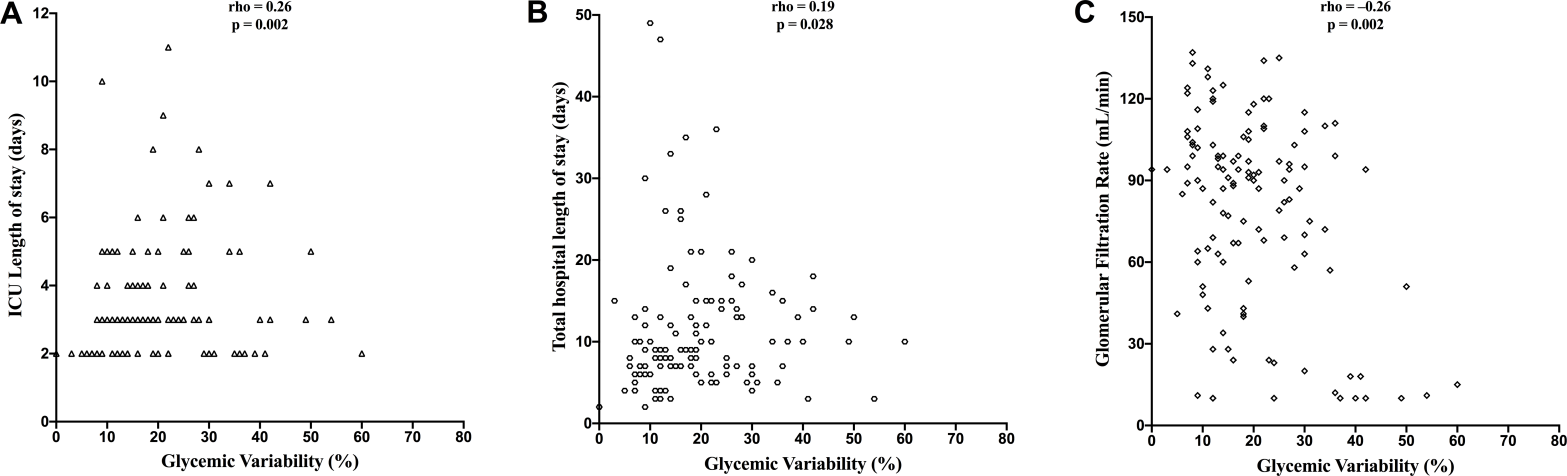
Correlations between glycemic variability and ICU length of stay, total hospital length of stay and glomerular filtration rate.

GV was positively correlated with total hospital LOS (rho = 0.19; p = 0.028) and ICU LOS (rho = 0.26; p = 0.002). GV also differed significantly according to mortality status (p = 0.049)and was negatively correlated with GFR (rho = –0.26; p = 0.002). No significant correlation was found between GV and BMI (rho = 0.08; p = 0.368) (**Figure 1**).

In contrast, MG did not correlate significantly with total hospital LOS (rho = 0.04; p = 0.635) and ICU LOS (rho = 0.06; p = 0.51),or in-hospital mortality (W = 8975, p=0.13). However, MG was significantly correlated with BMI (rho = 0.29; p = 0.001). After adjusting for BMI, the other correlations between MG and prognostic indicators such as hospital LOS, ICU LOS, and in-hospital mortality remained non-significant.

A threshold of GV>30% was selected *a priori* to define high GV. Based on the Cox model applied to mortality, a GV threshold > 30% was significantly associated with increased in-hospital mortality (HR = 6.04, 95% CI [1.18–30.90], p = 0.03).

### Comparison of biological and clinical profiles of diabetic and non-diabetic patients

MG was significantly higher among the 30 patients with known diabetes compared to those without a prior diabetes diagnosis (p < 0.0001). However, no significant differences were observed in GV (p = 0.33), hospital or ICU LOS (respectively p = 0.83 and p = 0.57), in-hospital mortality (p=0.11), or GFR (p=0.053) between groups.

## Discussion

In this cohort of 130 ICU patients, GV emerged as the primary predictor of adverse outcomes, whereas MG showed no significant associations. Higher GV was significantly associated with prolonged hospital and ICU LOS, reduced renal function, and increased in-hospital mortality. Notably, patients with GV >30% exhibited a six-fold higher risk of death. MG varied between ICU subunits, reaching its highest values in the cardiovascular ICU, but did not correlate with clinical outcomes. These findings highlight the superior prognostic value of GV over MG in critically ill patients.

Emerging evidence indicates that GV is associated with an increased risk of hypoglycemia, microvascular and macrovascular complications, cardiovascular risk, and mortality in outpatient populations with diabetes, independent of HbA1c levels (24–26). In hospitalized non-ICU patients, high GV has been linked to increased mortality (27) (28), prolonged hospital stays (29, 30), increased complication rates - such as infections, readmissions, and reoperations after lumbar spinal fusion (31) - and worse outcomes in cases of COPD exacerbation or community-acquired pneumonia (32). In acute coronary syndrome, a recent meta-analysis of 11 studies confirmed that elevated GV significantly increases cardiovascular risk (18). One study reported higher mortality in patients with diabetes with MG >140 mg/dL and GV >29% (28). In the ICU, multiple studies have examined GV in critically ill patients, both with and without diabetes (22, 33–39). GV predicted ICU mortality and was associated with extended ICU and overall hospital LOS, along with worse discharge outcomes (21, 22). In addition, analysis of the Medical Information Mart for Intensive Care IV (MIMIC-IV) database identified different GV thresholds above which mortality risk increases. Thresholds greater than 30%, 24.8%, 20.4% and 20% were found to be respectively associated with higher mortality rates in ICU patients with traumatic brain injury (22), acute kidney injury (35), aortic disease (36), and atrial fibrillation (37). In patients with hemorrhagic stroke, a threshold between 14 and 16% was reported (38). Although GV thresholds vary, these studies underscore GV as an independent prognostic marker for total hospital or ICU LOS (21, 22, 36–38) and ICU mortality (21, 22, 35–38). In our study, GV was positively associated with in-hospital mortality, with a GV threshold > 30% indicating a higher risk. Moreover, GV was negatively correlated with GFR, suggesting a potential link to renal dysfunction. Although causality cannot be inferred due to the cross-sectional design, these associations align with prior reports (21, 36–40). Plausible biological mechanisms support this relationship: in patients with and without type 2 diabetes, glucose fluctuations induce greater oxidative stress than sustained hyperglycemia (41). In hyperglycemic conditions, mitochondria, which are highly expressed in the kidney, undergo swelling, expansion, and structural damage, leading to the excessive production of reactive oxygen species within renal tubules (42). Experimental data also indicate that GV accelerates renal injury by inhibiting the AKT signaling pathway in diabetic rats (43). These mechanisms suggest that targeting GV could reduce morbidity, mortality while also preserving renal function. The correlation between GV and GFR may partly account for the association of GV with prolonged hospital stays and increased mortality.

Interestingly, MG showed no significant correlation with total hospital/ICU LOS, nor with in-hospital mortality, which may reflect the overall adequate glycemic control in our cohort. This aligns with previous studies reporting the limited benefit of strict glycemic control on cardiovascular events and mortality (12–14). Moreover, a significant positive correlation was observed between MG and BMI. This can be explained by obesity-related insulin resistance. Indeed, adipocytes can secrete TNF-alpha and free fatty acids, that impair metabolic regulation (44).

Comparing ICU subunits, the cardiovascular ICU had the highest median MG, reaching 140.7 mg/dL, which is at the upper limit of the current ADA recommendations (100–140 mg/dL) for patients in cardiovascular ICU settings (17). This may be attributed to higher diabetes prevalence, greater postoperative stress, older age, and higher BMI in this subgroup. In contrast, the medical ICU had the lowest average BMI, likely reflecting the hypercatabolic state of patients with acute or chronic systemic inflammation (45). It is also important to note that no significant difference in GV was observed across the three ICUs.

### Strengths and limitations of the study

Our study has several notable strengths. It is the first to simultaneously investigate the prognostic value of both GV and MG on total hospital and ICU LOS, while also comparing these parameters across different ICU subtypes. However, the study has some limitations. The sample size was relatively small, and the study period was limited to two months. In addition, the heterogeneity of ICU admissions and previous comorbidities precluded a detailed subgroup analysis.

## Conclusion

In conclusion, GV, rather than MG, emerged as a key predictor of adverse outcomes in ICU patients. It was associated with prolonged hospital and ICU stays, renal impairment, and increased in-hospital mortality. Notably, a GV threshold above 30% significantly elevated the risk of death. These findings underscore GV as an important therapeutic target in ICU management, highlighting the need for further prospective studies to establish a standardized GV threshold.

In clinical practice, continuous glucose monitoring (CGM) in ICU setting may offer significant benefits, particularly in preventing both hypoglycemic and hyperglycemic episodes among patients with diabetes. Recent ADA guidelines have begun recommending the use of digital monitoring devices in hospitalized patients (17).

## Data Availability

The raw data supporting the conclusions of this article will be made available by the authors, without undue reservation.
